# Translation, cross-cultural adaptation and psychometric validation of the Brazilian version of the dialysis patient-perceived exercise benefits and barriers scale

**DOI:** 10.1590/2175-8239-JBN-2025-0199en

**Published:** 2026-05-11

**Authors:** Graziella Alves da Silva, Cid André Fidelis de Paula Gomes, Thamiê Cristina Stella, Barbara G. Miura, Jing Zheng, Renato D. Foresto, Renata Kelly de Palma, Almir V. Dibai-Filho, Luciana Maria Malosá Sampaio

**Affiliations:** 1 Universidade Nove de Julho, Programa de Pós-Graduação em Ciências da Reabilitação, São Paulo, SP, Brazil.; 2 Guangdong Pharmaceutical University, School of Nursing, Guangzhou, China.; 3 Fundação Oswaldo Ramos, Hospital do Rim, São Paulo, SP, Brazil.; 4 Facultat de Ciències de la Salut de Manresa, Barcelona, España.; 5 Universidade Federal do Maranhão, Programa de Pós-Graduação em Educação Física, São Luís, MA, Brazil.

**Keywords:** Renal Insufficiency, Chronic, Renal Dialysis, Exercise, Surveys and Questionnaires

## Abstract

**Background:**

Chronic kidney disease (CKD) in dialysis patients compromises musculoskeletal health and reduces physical activity levels. The Dialysis Patient-Perceived Exercise Benefits and Barriers Scale (DPEBBS) was specifically developed to assess dialysis patients’ perceptions of exercise. This study aimed to translate, cross-culturally adapt, and evaluate the psychometric properties of the Brazilian version of the DPEBBS (EPAD).

**Methods:**

A cross-sectional study was conducted following the COSMIN guidelines. Psychometric properties assessed included reliability, internal consistency, and construct validity. Participants were recruited from the hemodialysis department of Unifesp. A total of 112 adults on dialysis completed the DPEBBS, the Short Form Health Survey-36 (SF-36), and underwent anthropometric evaluation. The interval between test–retest was one week. Descriptive and inferential analyses were performed to test validity and reliability.

**Results:**

The scale demonstrated high test–retest stability, with consistent mean scores across assessments. Internal consistency was strong, and reliability was supported by a low minimal detectable change and a high intraclass correlation coefficient. Convergent validity with the SF-36 Physical Functioning domain was weak but statistically significant (r = –0.326; p = 0.001), and the correlation with the General Health domain was weak and not statistically significant (r = –0.185; p = 0.052). Consistency analysis showed α = 0.885, ICC = 0.794, SEM = 4.96%, and demonstrated the absence of floor and ceiling effects.

**Conclusion:**

The EPAD showed robust validity and reliability for dialysis patients. Despite adequate reliability and validity, this study has limitations, including a single-center sample. The EPAD may support individualized exercise counseling and rehabilitation planning in hemodialysis units.

## Introduction

Chronic kidney disease (CKD) is defined as abnormalities in kidney structure or function lasting more than three months and is classified into five stages according to the level of glomerular filtration rate (GFR) reduction. In advanced stages, when GFR falls below 15 mL/min/1.73 m^2^, patients develop endstage renal disease and require renal replacement therapy (RRT), which includes kidney transplantation or dialysis^1,2^. Dialysis may be performed as either peritoneal dialysis or hemodialysis, with the choice of modality depending on the patient’s clinical condition and lifestyle rather than the superiority of one method over another^3^.

Currently, CKD is a significant public health issue in Brazil, with the prevalence of patients enrolled in dialysis programs increasing exponentially in recent years. The number of patients on dialysis more than doubled between 1994 and 2004, from 24,000 patients in 1994 to 59,153 patients in 2004, with the incidence of new patients growing by about 8% per year^4^. From 2017 to 2018, the number of patients with kidney disease with access to treatment increased by 98% in Brazil compared with the period from 2007 to 2009^5^. A comparison of Brazilian data with global statistics reinforces the relevance of policies aimed at the prevention and treatment of CKD, especially in the context of low- and middle-income countries, where the disease burden is disproportionately higher. CKD is also strongly associated with cardiovascular diseases, contributing to 1.4 million heart-related deaths and 25.3 million disability-adjusted life years (DALYs) due to impaired kidney function. This information highlights the need for greater global attention to CKD, particularly in regions such as Latin America, where the disease burden is higher than expected for the level of socioeconomic development^6^.

Chronic diseases are the leading cause of premature death in adults worldwide, particularly in low- and middle-income countries, where 80% of these deaths occur. It is estimated that in 2005, chronic diseases accounted for 35 million (60%) of a total of 58 million deaths from all causes^7,8^. Among these conditions, CKD is prominent, with systemic arterial hypertension, diabetes, and dyslipidemia among its main contributing factors^5^, emphasizing the importance of multidisciplinary follow-up for these patients. Among the various complications associated with CKD are physical disability, weakness, reduced quality of life, and an increased risk of death from cardiovascular events^9,10^.

Public spending associated with the chronic treatment of CKD in dialysis patients is also a concern, as there is significant utilization of healthcare resources, including consultations, hospitalizations, and renal replacement therapy (RRT)^1^.

It is well established that physical activity provides substantial benefits for patients with CKD, particularly those undergoing dialysis^11^. Regular exercise is associated with improvements in musculoskeletal health, physical function, fatigue, quality of life, and cardiorespiratory fitness and may also contribute to reduced cardiovascular morbidity and mortality^12^. Despite this robust body of evidence, most dialysis patients remain insufficiently active, and adherence to exercise and rehabilitation programs is consistently low^13^.

Multiple factors contribute to the low levels of physical activity observed in this population. Among the most frequently reported determinants are perceived barriers, such as fatigue, fear of injury, comorbidities, and physical limitations, as well as a lack of awareness or understanding of the specific benefits of exercise. In addition, insufficient counseling and encouragement from healthcare professionals may further limit engagement in physical activity^14,15^. Sociodemographic factors, including age and sex, as well as the type and context of physical activity, also play a significant role in influencing participation among adults in general and among dialysis patients in particular^16^.

Although these barriers and determinants are well recognized, their systematic assessment in clinical and research settings remains challenging. While several generic physical activity questionnaires are available, few instruments are specifically designed for and psychometrically validated in the dialysis population. This represents an important gap, as dialysis patients have unique clinical characteristics, treatment-related constraints, and perceptions that may not be adequately captured by generic tools. Therefore, the availability of valid and reliable instruments tailored to dialysis patients is essential for better understanding physical activity behavior, for identifying modifiable barriers, and supporting individualized exercise counseling and rehabilitation planning in this population^9,10,14^.

Different scales and questionnaires aim to measure physical activity levels in adults^17^, as well as establish effective proposals for activities with higher adherence rates, and this is no different for chronic kidney disease patients^18^. Currently, several scales and questionnaires evaluate physical activity levels and potential barriers faced by the adult population when engaging in physical activity; however, it is important that this evaluation be conducted specifically in patients with chronic kidney disease, considering their particularities. In this context, the tools available for such evaluation are considerably scarcer^19,20^.

Given the persistent low adherence to exercise among dialysis patients and the central role of perceived benefits and barriers in shaping physical activity behavior, the use of instruments that specifically capture these constructs is essential^21^. The Dialysis Patients’ Exercise Benefits and Barriers Scale (DPEBBS)^22^ was developed in China to assess dialysis patients’ perceptions of the benefits of exercise and the barriers that limit its practice, addressing dimensions that are directly relevant to this population and not fully captured by generic physical activity questionnaires. Therefore, the DPEBBS represents an appropriate and clinically meaningful tool for evaluating key determinants of exercise behavior in dialysis patients, supporting both research and individualized exercise counseling and rehabilitation planning.

Scales developed for other populations or in other languages need to be validated before they can be used safely and reproducibly in different languages and cultures. As the DPEBBS was originally developed in China, its translation, cross-cultural adaptation, and validation are required to ensure its conceptual equivalence, reliability, and validity for use in other linguistic and cultural contexts. Validation studies of available tools for various assessments have been effective in supporting the evaluation and treatment of patients across different health conditions^23^. This study aimed to translate and cross-culturally adapt the DPEBBS into Brazilian Portuguese and evaluate its psychometric properties in adult patients on hemodialysis.

## Methods

### Study Design and Participants

A cross-sectional study was conducted in which the DPEBBS, the SF-36, and anthropometric assessments were applied, following the COSMIN guidelines to ensure the reliable measurement of the scale’s properties. These guidelines provide different classifications based on sample size, with a sample of ≥100 participants being classified as “very good.”^24^ This classification aims to assess internal consistency, agreement, reliability, and convergent validity of a questionnaire undergoing validation and cross-cultural adaptation. Factor analysis was not performed in the present study because the factor structure of the DPEBBS has already been established in the original instrument through exploratory and confirmatory factor analyses. Given that our primary aim was cross-cultural adaptation and the assessment of reliability and validity, the previously validated structure was retained; therefore, a minimum sample size of 100 participants was considered adequate.

To be included in this study, participants needed to have a diagnosis of dialysis-dependent CKD, be ≥18 years old, and be clinically stable (i.e., absence of cardiovascular events, medication changes, or dialysis regimen modifications in the previous four weeks, and stable general health). Individuals who did not adhere to dialysis, as well as those with other diseases or physical conditions that prevented them from undergoing the assessments due to musculoskeletal or cognitive limitations, were excluded from the study. Patients were recruited from the Hemodialysis Department of the *Hospital do Rim* – Osvaldo Ramos Foundation – Federal University of São Paulo (Unifesp). After receiving an explanation of the study and its procedures and signing the Informed Consent Form (ICF), participants underwent anthropometric evaluation and completed the SF-36 and the DPEBBS.

### Translation and Cross-Cultural Adaptation

The translation and cross-cultural adaptation of the DPEBBS followed established international recommendations for the adaptation of health-related instruments. The original scale, developed in Chinese, was initially translated into Portuguese by a Brazilian physician fluent in Chinese, with clinical experience in dialysis care and familiarity with exercise-related concepts.

Subsequently, the Portuguese version was back-translated into Chinese by a native Chinese physician fluent in Portuguese, who was blinded to the original version of the instrument and had no access to its content. The back-translated version was compared with the original Chinese scale to assess semantic, idiomatic, and conceptual equivalence. A third translator, fluent in both Chinese and Portuguese and not affiliated with the healthcare field, evaluated any terms that might hinder patient understanding.

A pretest was conducted with 10 dialysis patients to evaluate clarity, comprehension, and cultural adequacy of the translated instrument. No difficulties in understanding the items or response options were identified, and no changes were required following the pretest. The pilot administration of the scale was carried out by physiotherapists, who also reported no difficulties in administering the instrument.

Finally, the back-translated version was sent to the School of Nursing at Sun Yat-sen University, the copyright holder of the DPEBBS, who suggested minor wording adjustments. These recommendations were incorporated into the final Portuguese version of the scale, which was subsequently applied to the full study sample. The scale was applied twice to the participants to assess test–retest reliability, with a one-week interval between assessments. This interval was short enough to avoid clinical changes but long enough to minimize recall of previous responses. Only clinically stable patients were reassessed for test–retest reliability, according to the predefined stability criteria.

### Statistical Analysis

Data analysis was performed using SPSS software (version 26.0; SPSS Inc.; Chicago, Illinois, USA). The Shapiro–Wilk test was used to determine data normality. In the descriptive analysis, parametric variables were expressed as mean and standard deviation (SD). For non-parametric variables, the median and interquartile range (IQR) were used, and categorical variables were expressed as absolute numbers, percentages, and frequencies. Internal consistency was tested using Cronbach’s alpha coefficient for the total questionnaire score. The index ranges from 0 to 1, and higher values indicate greater reliability of the questionnaire. Values between 0.75 and 0.95 were considered appropriate^25^.

Agreement was tested through the standard error of measurement (SEM) and the minimal detectable difference at 90% confidence (MDD90). MDD90 was calculated as (test score 1 - test score 2) / (√2 x SEM)^26,27^. SEM was considered very good if <5% of the total score, good if ≥5% and <10%, questionable if ≥10%, and doubtful if >20%^28^. Additionally, agreement was assessed using the Bland–Altman technique. Reliability was tested through the intraclass correlation coefficient (ICC), using the absolute agreement subtype for single measurements. The variance of each participant’s measurements, rather than the mean, was considered, along with the corresponding 95% confidence interval. The adopted classification was ICC ≤0.4: poor; 0.4 ≤ ICC ≤ 0.75: satisfactory; and ICC ≥0.75: excellent^29^. Convergent validity was analyzed using Pearson’s correlation for parametric variables and Spearman’s correlation for non-parametric variables, correlating the scores of the SF-36 domains with the total score of the DPEBBS. The correlation was characterized as follows: > 0.5, instruments with similar constructs; 0.3 to 0.5, instruments with related constructs; < 0.3, instruments with unrelated constructs^30^. Our hypothesis was that there would be a negative correlation between the questionnaires, with a magnitude <0.3. Ceiling and floor effects were tested by frequency and considered present if 15% or more of the patients achieved the maximum or minimum questionnaire score^28^.

## Results

A total of 118 patients were eligible for enrollment, and 112 were included in this study, with 6 patients excluded: 1 due to communication barriers (deafblindness), 1 due to neurological deficits, 2 due to cognitive impairment, 1 due to COVID-19, and 1 for not completing the second DPEBBS assessment.

The descriptive analysis revealed key characteristics of the participants. The mean age was 53.3 years, 61.6% were female, and most participants used a catheter for hemodialysis access (53.6%). The mean number of hospitalizations was 0.31 in the past year and 2.15 over the past five years. Additional descriptive data are presented in Table 1. The mean score in the first DPEBBS administration was 66.97 (SD 7.52), and in the second administration it was 66.96 (SD 7.71) (Table 2).

**Table 1:** General Characteristics

	Mean (SD)	Frequency (%)
Age (years)	53.27 (13.83)	
Female		69 (61.6)
Male		43 (38.4)
Catheter Dialysis		60 (53.6)
Fistula Dialysis		52 (46.4)
Height (m)	1.61 (0.10)	
BMI	24.13 (3.97)	
% Body Fat	31.66 (8.44)	
Hemoglobin (g/dL)	12.217 (1.77)	
Hematocrit (%)	37.86 (5.17)	
Platelets (/mm^3^)	198500.00 (92511.80)	
Creatinine (mg/dL)	10.09 (2.90)	

**Table 2:** Internal consistency, agreement, and reliability

	Test	Retest	Internal consistency	Agreement		Reliability
	Mean (SD)	Mean (SD)	Cronbach’s α	SEM	MDC90	ICC (95%)
Score	66.96 (7.52)	66.80 (7.71)	0.885	4.96%	0.02 (0.16)	0.794 (0.73 –0.85)
Question 1	2.87 (0.76)	2.97 (0.80)			–0.01 (–0.09)	
Question 2	3.14 (0.69)	3.10 (0.74)			0.005 (0.03)	
Question 3	3.14 (0.59)	3.20 (0.64)			–0.008 (–0.06)	
Question 4	3.30 (0.61)	3.34 (0.66)			–0.005 (–0.03)	
Question 5	2.98 (0.79)	2.89 (0.83)			0.01 (0.09)	
Question 6	3.11 (0.68)	3.16 (0.69)			–0.006 (–0.04)	
Question 7	3.19 (0.58)	3.26 (0.55)			–0.009 (–0.06)	
Question 8	1.82 (0.67)	1.79 (0.67)			0.005 (0.03)	
Question 9	2.58 (0.91)	2.52 (0.94)			0.008 (0.63)	
Question 10	2.84 (0.78)	2.94 (0.79)			–0.014 (–0.09)	
Question 11	2.74 (0.78)	2.66 (0.88)			0.012 (0.08)	
Question 12	2.66 (0.88)	2,48 (0,90)			0.02 (0.18)	
Question 13	3.16 (0.65)	3.15 (0.67)			0.001 (0.009)	
Question 14	2.25 (0.85)	2.20 (0.91)			0.006 (0.04)	
Question 15	2.56 (0.89)	2.51 (0.96)			0.007 (0.04)	
Question 16	3.27 (0.83)	3.36 (0.58)			–0.01 (–0.09)	
Question 17	2.75 (0.90)	2.71 (1.00)			0.006 (0.04)	
Question 18	2.71 (0.89)	2.62 (0.97)			0.01 (0.09)	
Question 19	2.09 (0.83)	2.02 (0.89)			0.008 (0.06)	
Question 20	3.13 (0.67)	3.21 (0.69)			–0.01 (–0.08)	
Question 21	2.55 (0.85)	2.59 (0.90)			–0.005 (–0,03)	
Question 22	3.14 (0.61)	3.19 (0.64)			–0.006 (–0.04)	
Question 23	2.55 (0.80)	2.71 (0.88)			–0.02 (–0.15)	
Question 24	2.33 (0.92)	2.24 (1.00)			0.01 (0.09)	

Abbreviations – SD: Standard deviation; SEM: Standard error of measurement; MDC90: Minimal detectable change at 90% confidence; D: Difference; ICC: Intraclass correlation coefficient; *p*: Significance; R: Pearson’s correlation coefficient.

Construct validity was assessed by examining correlations between the total DPEBBS score and the domains of the SF-36. Spearman’s correlation coefficients showed weak and predominantly nonsignificant associations across the SF-36 domains, including Physical Functioning (ρ = –0.177, p = 0.063), Role Physical (ρ = –0.205, p = 0.031), Role Emotional (ρ = –0.191, p = 0.045), Vitality (ρ = –0.059, p = 0.542), Mental Health (ρ = –0.076, p = 0.430), Social Functioning (ρ = –0.097, p = 0.311), Bodily Pain (ρ = –0.056, p = 0.559), General Health (ρ = –0.050, p = 0.601), and Health Transition (ρ = 0.008, p = 0.933) (Table 3).

**Table 3:** Validity with each SF-36 domain

SF–36 domain	ρ	p–value
Physical Functioning	–0.177	0.063
Role Physical	–0.205*	0.031
Role Emotional	–0.191*	0.045
Vitality	–0.059	0.542
Mental Health	–0.076	0.430
Social Functioning	–0.097	0.311
Bodily Pain	–0.056	0.559
General Health	–0.050	0.601
Health Transition	0.008	0.933

Notes – ρ = Spearman’s correlation coefficient.

* p < 0.05.

Convergent and divergent validity hypotheses were tested using Spearman’s correlation between the DPEBBS total score and the Physical Functioning and General Health domains of the SF-36. A weak but statistically significant negative correlation was observed with Physical Functioning (ρ = –0.326, p = 0.001), supporting convergent validity, whereas the correlation with General Health was weak and not statistically significant (ρ = –0.185, p = 0.052), indicating the absence of divergent validity. Although the *a priori* hypothesis defined convergent validity as a weak negative correlation (|ρ| < 0.30), the observed correlation with Physical Functioning (ρ = –0.326) was slightly higher than expected. Nevertheless, the magnitude of the association remains weak and compatible with the interpretation of related yet distinct constructs, in accordance with the COSMIN recommendations.

The SF-36 scoring analysis revealed the following mean results (Table 4): Physical functioning: 96% (SD 3.38); Role physical: 42% (SD 0.40); Role emotional: 47% (SD 0.43); Vitality: 53% (SD 0.23); Mental health: 68% (SD 0.23); Social functioning: 66% (SD 0.31); Bodily pain: 66% (SD 0.29); General health: 45% (SD 0.18); Health transition: 54% (SD 0.27).

**Table 4:** SF-36 results

	Score (SD)
Physical Functioning	96% (3.38)
Role Physical	42% (0.40)
Role Emotional	47% (0.43)
Vitality	53% (0.23)
Mental Health	68% (0.23)
Social Functioning	66% (0.31)
Bodily Pain	66% (0.29)
General Health	45% (0.18)
Health Transition	54% (0.27)

Abbreviations – SD: Standard deviation.

**Figure 1:**
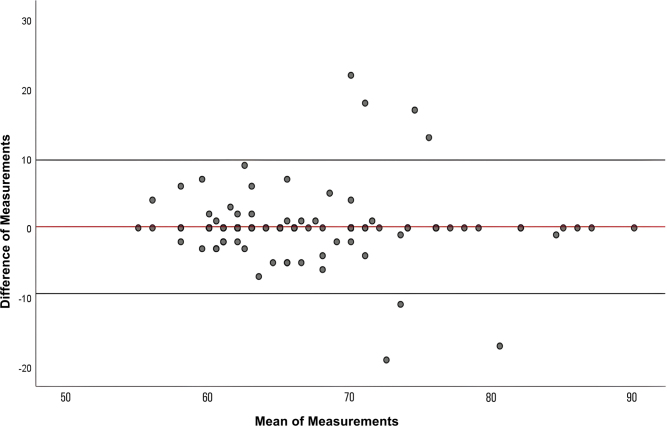
Bland–Altman graphic.

The DPEBBS showed high internal consistency (Cronbach’s α = 0.885), very good measurement error (SEM = 4.96% of the total score), and good test–retest reliability (ICC = 0.794, 95% CI 0.73–0.85), with no floor or ceiling effects observed. These results are also illustrated in the Bland–Altman plot (Figure 1). The intraclass correlation coefficient for single measures was 0.794 (95% CI 0.73–0.85), indicating good reliability of the data (Table 2). No ceiling or floor effects were observed.

## Discussion

Chronic kidney disease (CKD) represents a significant burden on public health systems worldwide, with its prevalence steadily increasing, especially in low- and middle-income countries such as Brazil^31^. The rise in the number of patients enrolled in dialysis programs highlights the urgent need for effective management strategies tailored to this population^32^. In this study, we aimed to address the shortage of validated tools specifically designed to assess physical activity levels and perceived barriers among CKD patients on dialysis. The Dialysis Patient-Perceived Exercise Benefits and Barriers Scale (DPEBBS) was chosen for validation due to its potential to provide valuable insights into the factors that influence participation in physical activity among dialysis patients. Through rigorous processes of translation and cross-cultural adaptation following the COSMIN guidelines, we ensured the reliability and validity of the scale for use in the Brazilian context.

This study faced significant challenges in translating and culturally adapting a scale from Chinese to Brazilian Portuguese. The linguistic and cultural differences between the languages required meticulous semantic analysis to ensure the equivalence of terms and concepts. Additionally, cultural adaptation was essential to ensure that the scale items accurately reflected the experiences and perceptions of Brazilian patients. The validation process involved a pilot test and feedback from healthcare professionals and patients, resulting in refinements to improve the comprehension and applicability of the scale in the new population. The expertise of translators specialized in medical terminology was crucial in mitigating linguistic inconsistencies and ensuring the consistency of the translation across all scale items. As a result of these efforts, a validated translated version of the scale was obtained, which proved to be effective and reliable for assessing Brazilian patients, thus contributing to a better understanding and treatment of their specific needs.

Data analysis revealed that the scale demonstrated positive characteristics in terms of stability and consistency. The mean test scores were 66.96, while the mean retest scores were 66.80. These similar means indicate good stability of scores over time. The internal consistency of the instrument, measured by Cronbach’s α, was 0.885. This high value suggests that the items are cohesive and consistently measure the same construct. The standard error of measurement (SEM) was 4.96%, which indicates reliable mean scores with little variation. The reliability of the scale was also confirmed by the minimal detectable change at 90% confidence (MDC90) between the test–retest, which was 0.02 (0.16). This indicates that the variation in participants’ responses between measurements was minimal, further reinforcing the instrument’s reliability.

The intraclass correlation coefficient (ICC) was 0.794, with a 95% confidence interval between 0.73 and 0.85. This index confirms good agreement between the measurements, suggesting that the scale is stable when comparing the same individuals’ scores at different times. Regarding validity, most of the correlations found between the DPEBBS and the SF-36 domains were below 0.3, indicating that the constructs are not strongly related. Only the “Role Physical” and “Role Emotional” domains showed weak, statistically significant negative correlations, but still within the category of unrelated constructs, according to the COSMIN classification.

When analyzing the specific items of the scale, the mean responses varied slightly between the test–retest, but the correlations showed minimal variation, indicating high consistency and stability in the responses. In summary, the statistical analysis revealed good internal consistency, reliability, and convergent validity, as well as adequate divergent validity. These characteristics reinforce the robustness of the instrument in assessing the constructs it is intended to measure.

The descriptive data of the participants provide a comprehensive overview of the demographic and clinical characteristics of the sample. The participants’ mean age was 53.27 (±13.83) years, with a mean BMI of 24.13 (±3.97). These values are consistent with typical populations of patients on hemodialysis, despite demographic variations within this population^33,34^. Sex distribution revealed a higher prevalence of female participants at 61.6%, which should be considered when generalizing the results to broader populations^35^. The presented biochemical parameters (such as hemoglobin, hematocrit, creatinine, urea, potassium, phosphorus, calcium, sodium, ferritin, and iron) were within the expected ranges for hemodialysis patients, reflecting the complexity and challenges of managing this condition^36^. The mean number of hospitalizations in recent years is a relevant metric that highlights the disease’s burden and the need for ongoing interventions to maintain the health of these patients, with an average of 0.31 (±0.54) hospitalizations in the last year and 2.15 (±1.93) over the last five years^37,38^.

The analysis of Short Form Health Survey-36 (SF-36) scores revealed notable impairments in various domains of health-related quality of life among the study participants. Role physical, role emotional, and reduced vitality were particularly pronounced, highlighting the multifaceted impact of CKD on patients’ well-being^39^. These findings underscore the importance of addressing not only physical barriers but also psychosocial factors that may influence engagement in physical activity among dialysis patients.

Compared with the original study by Zheng et al., the population in the present study showed some differences that may have influenced the validity values. In the original study, the population was mostly male, and the primary dialysis access was an arteriovenous fistula, whereas in this study, the majority of the participants were female, and the primary dialysis access was a catheter^22^.

Regarding the results obtained when comparing quality of life and the perception of barriers and benefits that dialysis patients have regarding physical activity, our study found results similar to those of Zheng et al., with the main issue being the low quality of life in the evaluated population. However, these individuals still recognized the benefits of physical activity, suggesting that low adherence to exercise and rehabilitation programs may be associated with other factors such as fatigue or fear of injury^9^. The psychometric indices observed for the DPEBBS in this study are also comparable to those reported in previous studies using the DPEBBS and other patient-reported outcome measures in hemodialysis populations^12^. In the study by Ghafourifard et al., the DPEBBS demonstrated satisfactory internal consistency and meaningful associations with patient-reported outcomes, supporting its use in clinical research. Similarly, the DPEBBS showed high internal consistency, good test–retest reliability, and acceptable measurement error, indicating stable and reproducible scores over time. These reliability indices are in line with those commonly reported for widely used instruments in dialysis care, such as the KDQOL-SF and the SF-36, which typically present adequate internal consistency and reproducibility despite measuring broader constructs that are not specific to exercise. Therefore, the DPEBBS demonstrates psychometric performance comparable to established scales used in dialysis settings while offering the advantage of specifically capturing patients’ perceptions of exercise-related benefits and barriers, a construct directly relevant to the planning and evaluation of physical activity interventions in hemodialysis care.

The findings of the present study are largely consistent with those reported by Lightfoot et al. (2021)^14^, who investigated perceptions of exercise benefits and barriers in a large sample of patients undergoing hemodialysis and peritoneal dialysis using the DPEBBS. In both studies, fatigue emerged as one of the most frequently reported barriers, confirming its central role as a limiting factor for exercise participation in this population. Similarly, benefits related to improvements in quality of life, mood, and physical functioning were widely recognized by patients across both contexts, suggesting a consistent perception of the positive effects of exercise. However, differences in the frequency of certain barriers, such as perceptions related to comorbidities and the impact of exercise on family life, were observed between the studies, possibly reflecting variations in clinical profiles, care settings, and study design. Despite these differences, the overall response patterns indicate that patients undergoing dialysis share similar perceptions regarding the main benefits and obstacles to exercise, reinforcing the need for intervention strategies that specifically address fatigue and emphasize benefits perceived as clinically meaningful by the patients themselves.

Wingood et al. highlighted substantial heterogeneity in the assessment of perceived physical activity barriers among adults aged 50 years and older and emphasized the need for populationspecific instruments^21^. Although 33 different tools were identified in the review, most were validated in a single study, and none were developed specifically for patients undergoing dialysis. In this context, the present study fills an important gap by providing a validated Brazilian version of an instrument tailored to the dialysis population. Unlike generic scales such as the Exercise Benefits/Barriers Scale (EBBS), the DPEBBS addresses benefits and barriers directly related to the dialysis treatment context, which may explain the weak but theoretically coherent correlations observed with the SF-36 domains. These results support the view that instruments focused on exercise barriers assess constructs related to, but distinct from, general quality of life and underscore the importance of robust psychometric evaluation in specific clinical populations, particularly in low- and middle-income countries with a high burden of chronic kidney disease.

Future research should focus on evaluating the responsiveness of the DPEBBS in hemodialysis rehabilitation trials, particularly in studies aimed at promoting physical activity and exercise adherence. Longitudinal investigations are also needed to establish clinically meaningful cutoff points, such as scores associated with low exercise participation or poor adherence to rehabilitation programs. Furthermore, the validation of alternative versions of the scale, including electronic formats and adaptations to other languages, may further expand its applicability across different clinical and cultural settings.

### Implications for Physiotherapy Practice

The findings of this study suggest that the DPEBBS can be incorporated into clinical practice as a decision-support tool, particularly in the rehabilitation of patients undergoing hemodialysis. Its application enables the identification of patients with a higher perception of barriers to physical activity, facilitating behavioral risk stratification and prioritization of interventions.

Additionally, the instrument may support the development of individualized exercise strategies tailored to patients’ specific needs and perceptions, contributing to more patient-centered approaches. In this context, its use may optimize therapeutic planning, particularly in intradialytic or home-based exercise programs, by integrating both physical and psychosocial factors related to exercise adherence.

### Limitations

This study has several limitations that should be considered when interpreting the findings. First, it was conducted in a single hemodialysis center, which may limit the generalizability of the results to other populations and healthcare settings.

Furthermore, the predominance of female participants may have influenced the findings, given potential sex-related differences in the perception of exercise-related benefits and barriers.

The use of the SF-36 as a measure of construct validity, although widely accepted, may not specifically capture exercise-related constructs, which may partially explain the low-magnitude correlations observed.

In addition, the lack of responsiveness assessment limits the understanding of the instrument’s ability to detect changes over time. Finally, the absence of exploratory or confirmatory factor analysis restricts the evaluation of the scale’s dimensional structure within the Brazilian population.

## Conclusion

The Brazilian version received the nomenclature “*Escala de Percepção da Atividade Física em Diálise* (EPAD).”

The EPAD demonstrated adequate validity and reliability for use in dialysis patients, representing a robust instrument for assessing perceived benefits and barriers to physical activity. Despite its satisfactory psychometric properties, limitations such as the single-center design should be considered when interpreting the findings. Nevertheless, the EPAD shows potential for clinical application, supporting individualized exercise counseling and rehabilitation planning in hemodialysis settings, and contributing to more patient-centered care strategies.

## Data Availability

The data that support the findings of this study are available from the corresponding author upon reasonable request.

## Supplementary Material

The following online material is available for this article:

Brazilian Portuguese version of the DPEBBS.
