# Supplementary material for: Translation, cross-cultural adaptation and psychometric validation of the Brazilian version of the dialysis patient-perceived exercise benefits and barriers scale
Source: J Bras Nefrol. 2026 May 11;48(3):e20250199. doi: 10.1590/2175-8239-JBN-2025-0199en (PMC13178697; doi:10.1590/2175-8239-JBN-2025-0199en)
Supplement: Supplementary file 1 [file 2175-8239-jbn-48-3-e20250199-suppl1.pdf]

Brazilian Journal of Nephrology
https://doi.org/10.1590/2175-8239-JBN-2025-0199en

Supplementary Material to "Translation, cross-cultural adaptation and psychometric validation of the Brazilian version of the Dialysis Patient-Perceived Exercise Benefits and Barriers Scale"

Brazilian – Portuguese Version of the DPEBBS

Escala de Percepção de Benefícios e Barreiras à Atividade Física para Pacientes em Diálise

	Concordo Plenamente	Concordo	Discordo	Discordo Plenamente
1. Exercícios podem ajudar a reduzir todos os tipos de gastos médicos que tenho				
2. Exercícios me ajudam a reduzir a dor no corpo				
3. Exercícios podem adiar o declínio na função corporal				
4. Exercícios previnem a atrofia muscular				
5. Cansaço frequente impede minha participação no exercício				
6. Exercício melhora meu humor				
7. Exercícios melhoram as doenças ósseas				
8. Exercício é prejudicial à saúde física de pacientes dialíticos				
9. Tenho medo de cair durante exercícios				
10. Exercícios aumentam o meu apetite				
11. Frequentemente a fadiga muscular dos membros inferiores impede minha participação no exercício				
12. Me falta conhecimento sobre os benefícios do exercícios				

13. Exercício me ajuda a ter uma vida otimista e ativa				
14. Exercícios não são adequados para mim já que tenho outras comorbidades				
15. A dor no corpo me impede de me exercitar.				
16. Exercícios melhoram minha qualidade de vida				
17. Me falta entendimento sobre o que devo saber para fazer exercícios				
18. Me preocupo que fazer exercícios me faça sentir sede				
19. Exercícios não são adequados para mim já que tenho doença renal				
20. Exercícios podem manter meu peso corporal em um nível estável				
21. Me preocupo que exercícios possam afetar minha fístula arteriovenosa				
22. Exercícios ajudam melhorar minhas habilidades de autocuidado				
23. Exercícios me impedirão de ter outras doenças (ex.: gripe)				
24. O exercício ao ar livre aumenta a sobrecarga da minha família porque preciso da companhia deles quando saio para me exercitar				

Quais outros benefícios você acha que o exercício tem?

....

Quais outros fatores você acha que podem impedir sua participação em exercícios?

....
