# Supplementary material for: Translation, cross-cultural adaptation and psychometric validation of the Brazilian version of the dialysis patient-perceived exercise benefits and barriers scale
Source: J Bras Nefrol. 2026 May 11;48(3):e20250199. doi: 10.1590/2175-8239-JBN-2025-0199en (PMC13178697; doi:10.1590/2175-8239-JBN-2025-0199en)
Supplement: Translation

# Translation: Tradução, adaptação transcultural e validação psicométrica da versão brasileira da escala de benefícios e barreiras ao exercício percebidos por pacientes em diálise

**DOI:** 10.1590/2175-8239-JBN-2025-0199pt

**Authors:** Graziella Alves da Silva, Cid André Fidelis de Paula Gomes, Thamiê Cristina Stella, Barbara G. Miura, Jing Zheng, Renato D. Foresto, Renata Kelly de Palma, Almir V. Dibai-Filho, Luciana Maria Malosá Sampaio

**Affiliations:** 1 Universidade Nove de Julho, Programa de Pós-Graduação em Ciências da Reabilitação, São Paulo, SP, Brasil.; 2 Guangdong Pharmaceutical University, School of Nursing, Guangzhou, China.; 3 Fundação Oswaldo Ramos, Hospital do Rim, São Paulo, SP, Brasil.; 4 Facultat de Ciències de la Salut de Manresa, Barcelona, España.; 5 Universidade Federal do Maranhão, Programa de Pós-Graduação em Educação Física, São Luís, MA, Brasil.

**Keywords:** Insuficiência Renal Crônica, Diálise Renal, Exercício Físico, Inquéritos e Questionários

## Abstract

**Introdução:**

A doença renal crônica (DRC) em diálise compromete a saúde musculoesquelética e reduz os níveis de atividade física. A *Dialysis Patient-Perceived Exercise Benefits and Barriers Scale* (DPEBBS) foi desenvolvida especificamente para avaliar as percepções dos pacientes em diálise sobre o exercício. Este estudo teve como objetivo traduzir, adaptar transculturalmente e avaliar as propriedades psicométricas da versão brasileira da DPEBBS (EPAD).

**Métodos:**

Foi conduzido um estudo transversal, seguindo as diretrizes do COSMIN. As propriedades psicométricas avaliadas incluíram confiabilidade, consistência interna e validade de construto. Os participantes foram recrutados no setor de hemodiálise da Unifesp. Um total de 112 adultos em diálise respondeu à DPEBBS, ao *Short Form Health Survey-36* (SF-36), e foi submetido à avaliação antropométrica. O intervalo entre o teste e o reteste foi de uma semana. Análises descritivas e inferenciais foram realizadas para testar a validade e a confiabilidade.

**Resultados:**

A escala demonstrou alta estabilidade teste–reteste, com médias consistentes entre as avaliações. A consistência interna foi alta, e a confiabilidade foi sustentada por uma mínima mudança detectável baixa e por um alto coeficiente de correlação intraclasse. A validade convergente com o domínio Capacidade Funcional do SF-36 foi fraca, porém estatisticamente significativa (r = –0,326; p = 0,001), enquanto a correlação com o domínio Estado Geral de Saúde foi fraca e não estatisticamente significativa (r = –0,185; p = 0,052). A análise de consistência mostrou α = 0,885, ICC = 0,794, SEM = 4,96%, e demonstrou ausência de efeitos de piso e teto.

**Conclusão:**

A EPAD apresentou validade e confiabilidade robustas para pacientes em diálise. Apesar da adequada confiabilidade e validade, este estudo apresenta limitações, incluindo uma amostra de centro único. A EPAD pode apoiar o aconselhamento individualizado para o exercício e o planejamento da reabilitação em unidades de hemodiálise.

## Introdução

A doença renal crônica (DRC) é definida como anormalidades na estrutura ou na função renal, com duração superior a três meses, sendo classificada em cinco estágios, de acordo com o nível de redução da taxa de filtração glomerular (TFG). Nos estágios avançados, quando a TFG cai abaixo de 15 mL/min/1,73 m^2^, os pacientes evoluem para doença renal em estágio terminal e necessitam de terapia renal substitutiva (TRS), que inclui o transplante renal ou a diálise^1,2^. A diálise pode ser realizada por meio de diálise peritoneal ou hemodiálise, sendo a escolha da modalidade dependente da condição clínica e do estilo de vida e não da superioridade de um método em relação ao outro^3^.

Atualmente, a DRC constitui um importante problema de saúde pública no Brasil, com aumento exponencial da prevalência de pacientes inseridos em programas de diálise nos últimos anos. O número de pacientes em diálise mais que dobrou entre 1994 e 2004, passando de 24.000 pacientes em 1994 para 59.153 em 2004, com crescimento anual da incidência de novos pacientes em torno de 8%^4^. Entre 2017 e 2018, o número de pacientes renais com acesso ao tratamento aumentou em 98% no Brasil em comparação ao período de 2007 a 2009^5^. A comparação dos dados brasileiros com estatísticas globais reforça a relevância de políticas voltadas à prevenção e ao tratamento da DRC, especialmente no contexto de países de baixa e média renda, onde a carga da doença é desproporcionalmente maior. A DRC também está fortemente associada às doenças cardiovasculares, contribuindo para 1,4 milhão de óbitos relacionados ao coração e 25,3 milhões de anos de vida ajustados por incapacidade (*Disability-Adjusted Life Years* – DALYs) atribuídos à disfunção renal. Essas informações evidenciam a necessidade de maior atenção global à DRC, particularmente em regiões como a América Latina, onde a carga da doença é superior à esperada para o nível de desenvolvimento socioeconômico^6^.

As doenças crônicas são a principal causa de morte prematura em adultos em todo o mundo, especialmente em países de baixa e média renda, onde ocorrem 80% desses óbitos. Estima-se que, em 2005, as doenças crônicas foram responsáveis por 35 milhões (60%) de um total de 58 milhões de óbitos por todas as causas^7,8^. Entre elas, destaca-se a doença renal crônica, cujos principais fatores desencadeantes são a hipertensão arterial sistêmica, o diabetes e a dislipidemia^5^, reforçando a importância do acompanhamento multidisciplinar desses pacientes. Entre as diversas complicações associadas à DRC estão a incapacidade física, a fraqueza, a redução da qualidade de vida e o aumento do risco de morte por eventos cardiovasculares^9,10^.

Os gastos públicos associados ao tratamento crônico da DRC em diálise também representam uma preocupação, uma vez que há grande utilização de recursos em saúde, incluindo consultas, hospitalizações e terapia renal substitutiva (TRS)^1^.

Está bem estabelecido que a atividade física proporciona benefícios substanciais para pacientes com doença renal crônica, particularmente aqueles em tratamento dialítico^11^. O exercício regular está associado a melhorias na saúde musculoesquelética, na função física, na fadiga, na qualidade de vida e na aptidão cardiorrespiratória, podendo também contribuir para a redução da morbimortalidade cardiovascular^12^. Apesar desse robusto corpo de evidências, a maioria dos pacientes em diálise permanece insuficientemente ativa, e a adesão a programas de exercício e reabilitação é consistentemente baixa^13^.

Diversos fatores contribuem para os baixos níveis de atividade física observados nessa população. Entre os determinantes mais frequentemente relatados estão as barreiras percebidas, como fadiga, medo de lesões, comorbidades e limitações físicas, além da falta de conhecimento ou compreensão sobre os benefícios específicos do exercício. Adicionalmente, o aconselhamento e o incentivo insuficientes por parte dos profissionais de saúde podem limitar ainda mais o engajamento em atividades físicas^14,15^. Fatores sociodemográficos, como idade e sexo, bem como o tipo e o contexto da atividade física, também desempenham papel importante na participação de adultos em geral e de pacientes em diálise em particular^16^.

Embora essas barreiras e determinantes sejam amplamente reconhecidos, sua avaliação sistemática em contextos clínicos e de pesquisa ainda representa um desafio. Apesar da disponibilidade de diversos questionários genéricos de atividade física^17^, poucos instrumentos foram especificamente desenvolvidos e validados psicometricamente para a população em diálise. Isso representa uma lacuna importante, uma vez que pacientes em diálise apresentam características clínicas, restrições relacionadas ao tratamento e percepções singulares que podem não ser adequadamente captadas por instrumentos genéricos. Dessa forma, a disponibilidade de instrumentos válidos e confiáveis, específicos para pacientes em diálise, é essencial para melhor compreender o comportamento em relação à atividade física e identificar barreiras modificáveis. Além disso, esses instrumentos podem apoiar o aconselhamento individualizado para o exercício e o planejamento da reabilitação nessa população^9,10,14^.

Diferentes escalas e questionários têm como objetivo mensurar os níveis de atividade física em adultos, além de estabelecer propostas eficazes de atividades com maiores taxas de adesão, e isso não é diferente para pacientes com doença renal crônica^18^. Atualmente, diversas escalas e questionários avaliam os níveis de atividade física e as barreiras potenciais enfrentadas pela população adulta para a prática de atividades físicas; contudo, é fundamental que essa avaliação seja realizada especificamente em pacientes com DRC, considerando suas particularidades. Nesse contexto, os instrumentos disponíveis para essa finalidade são muito mais escassos^19,20^.

Diante da persistente baixa adesão ao exercício entre pacientes em diálise e do papel central dos benefícios e barreiras percebidos na determinação do comportamento em relação à atividade física, o uso de instrumentos que capturem especificamente esses construtos é essencial^21^. A *Dialysis Patients’ Exercise Benefits and Barriers Scale* (DPEBBS)^22^ foi desenvolvida na China para avaliar as percepções de pacientes em diálise quanto aos benefícios do exercício e às barreiras que limitam sua prática, abordando dimensões diretamente relevantes para essa população e não plenamente contempladas por questionários genéricos de atividade física. Assim, a DPEBBS representa uma ferramenta apropriada e clinicamente relevante para avaliar determinantes-chave do comportamento de exercício em pacientes em diálise, apoiando tanto a pesquisa quanto o aconselhamento individualizado para o exercício e o planejamento da reabilitação.

Escalas desenvolvidas para outras populações ou em outros idiomas precisam ser validadas para que possam ser utilizadas de forma segura e reprodutível quando aplicadas em diferentes línguas e culturas. Como a DPEBBS foi originalmente desenvolvida na China, são necessárias a tradução, a adaptação transcultural e a validação para garantir sua equivalência conceitual, confiabilidade e validade para uso em diferentes contextos linguísticos e culturais. Estudos de validação de instrumentos disponíveis para diversas avaliações têm se mostrado eficazes no auxílio à avaliação e ao tratamento de pacientes com diferentes condições de saúde e doença^23^. Este estudo teve como objetivo traduzir e adaptar transculturalmente a DPEBBS para o português brasileiro e avaliar suas propriedades psicométricas em pacientes adultos em hemodiálise.

## Métodos

### Desenho do Estudo e Participantes

Foi conduzido um estudo transversal por meio da aplicação da DPEBBS, do SF-36 e de uma avaliação antropométrica, seguindo as diretrizes do COSMIN para garantir a mensuração confiável das propriedades da escala. Essas diretrizes fornecem diferentes classificações com base no tamanho amostral, sendo uma amostra com ≥100 participantes classificada como “*very good*”.^24^ Essa classificação visa avaliar a consistência interna, a concordância, a confiabilidade e a validade convergente de um questionário em processo de validação e adaptação transcultural.

A análise fatorial não foi realizada no presente estudo porque a estrutura fatorial da DPEBBS já foi estabelecida no instrumento original por meio de análises fatoriais exploratória e confirmatória. Considerando que o objetivo principal foi a adaptação transcultural e a avaliação da confiabilidade e validade, a estrutura previamente validada foi mantida; portanto, um tamanho amostral mínimo de 100 participantes foi considerado adequado.

Para inclusão no estudo, os participantes deveriam ter diagnóstico de DRC dependente de diálise, idade lise, idade 18 anos e estar clinicamente estáveis (ausência de eventos cardiovasculares, mudanças na medicação ou no regime dialítico nas quatro semanas anteriores e estado geral de saúde estável). Foram excluídos indivíduos que não aderiam à diálise, bem como aqueles com outras doenças ou condições físicas que impedissem a realização dos testes e exames devido a limitações musculoesqueléticas ou cognitivas.

Os pacientes foram recrutados no Departamento de Hemodiálise do Hospital Rim – Fundação Oswaldo Ramos – Universidade Federal de São Paulo (Unifesp). Após a explicação do estudo e dos procedimentos e a assinatura do Termo de Consentimento Livre e Esclarecido (TCLE), os participantes foram submetidos à avaliação antropométrica e preencheram o SF-36 e a DPEBBS.

### Tradução e Adaptação Transcultural

A tradução e a adaptação transcultural da DPEBBS seguiram as recomendações internacionais estabelecidas para a adaptação de instrumentos relacionados à saúde. A escala original, desenvolvida em chinês, foi inicialmente traduzida para o português por um médico brasileiro fluente em chinês, com experiência clínica em cuidados dialíticos e familiaridade com conceitos relacionados ao exercício físico.

Subsequentemente, a versão em português foi retrotraduzida para o chinês por um médico chinês nativo fluente em português, que estava cego à versão original do instrumento e não teve acesso ao seu conteúdo. A versão retrotraduzida foi comparada com a escala original em chinês para avaliar a equivalência semântica, idiomática e conceitual. Um terceiro tradutor, fluente em chinês e português e sem vínculo com a área da saúde, avaliou termos que pudessem dificultar a compreensão pelos pacientes.

Foi realizado um pré-teste com 10 pacientes em diálise para avaliar clareza, compreensão e adequação cultural do instrumento traduzido. Não foram identificadas dificuldades na compreensão dos itens ou das opções de resposta, e nenhuma modificação foi necessária após o pré-teste. A aplicação piloto da escala foi realizada por fisioterapeutas, que também relataram não haver dificuldades na administração do instrumento.

Por fim, a versão retrotraduzida foi enviada à *School of Nursing da Sun Yat-sen University*, detentora dos direitos autorais da DPEBBS, que sugeriu pequenos ajustes na redação. Essas recomendações foram incorporadas à versão final da escala em português, que foi posteriormente aplicada à amostra total do estudo.

A escala foi aplicada duas vezes aos participantes para avaliação da confiabilidade teste-reteste, com intervalo de uma semana entre as avaliações. Esse intervalo é suficientemente curto para evitar mudanças clínicas, mas longo o suficiente para minimizar a recordação das respostas anteriores. Apenas pacientes clinicamente estáveis foram reavaliados para a confiabilidade teste-reteste, de acordo com os critérios de estabilidade previamente definidos.

### Análise Estatística

A análise dos dados foi realizada utilizando o software SPSS (versão 26.0; SPSS Inc.; Chicago, Illinois, EUA). O teste de *Shapiro–Wilk* foi utilizado para determinar a normalidade dos dados. Na análise descritiva, as variáveis paramétricas foram expressas como média e desvio-padrão (DP). Para variáveis não paramétricas, foram utilizadas mediana e intervalo interquartil, e as variáveis categóricas foram expressas em números absolutos, porcentagens e frequências.

A consistência interna foi testada por meio do coeficiente alfa de *Cronbach* para o escore total do questionário. O índice varia de 0 a 1, sendo valores mais elevados indicativos de maior confiabilidade do questionário. Valores entre 0,75 e 0,95 foram considerados apropriados^25^.

A concordância foi testada por meio do erro padrão de medição (SEM) e da diferença mínima detectável com 90% de confiança (MDD90). A MDD90 foi calculada como: (escore do teste 1 - escore do teste 2) / (√2 × SEM)^26,27^. O SEM foi considerado muito bom se <5% do escore total, bom se ≥5% e <10%, questionável se ≥10% e duvidoso se >20%^28^.

Adicionalmente, a concordância do instrumento foi avaliada por meio da técnica de *Bland–Altman*. A confiabilidade foi testada utilizando o coeficiente de correlação intraclasse (ICC), subtipo concordância absoluta para medidas únicas. Foi considerada a variância das medidas de cada participante, e não a média, juntamente com o respectivo intervalo de confiança de 95%. A classificação adotada foi: ICC ≤0,4: fraca; 0,4 ≤ ICC ≤ 0,75: satisfatória; e ICC ≥0,75: excelente^29^.

A validade convergente foi analisada utilizando a correlação de *Pearson* para variáveis paramétricas e a correlação de *Spearman* para variáveis não paramétricas, correlacionando os escores dos domínios do SF-36 com o escore total da DPEBBS. A correlação foi caracterizada como: >0,5, instrumentos com construtos semelhantes; 0,3 a 0,5, instrumentos com construtos relacionados; <0,3, instrumentos com construtos não relacionados^30^. A hipótese estabelecida foi a de existência de correlação negativa entre os questionários, com magnitude <0,3.

Os efeitos teto e piso foram testados por frequência e considerados presentes se 15% ou mais dos pacientes atingissem o escore máximo ou mínimo do questionário^28^.

## Resultados

Um total de 118 pacientes foi considerado elegível para participação, dos quais 112 foram incluídos neste estudo, com 6 exclusões: 1 devido a barreiras de comunicação (surdez e cegueira), 1 por déficits neurológicos, 2 por comprometimento cognitivo, 1 por COVID-19 e 1 por não ter completado a segunda aplicação da DPEBBS.

A análise descritiva revelou características-chave dos participantes. A idade média foi de 53,3 anos, 61,6% eram do sexo feminino e a maioria utilizava cateter como acesso para hemodiálise (53,6%). O número médio de hospitalizações foi de 0,31 no último ano e de 2,15 nos últimos cinco anos. Dados descritivos adicionais estão apresentados na Tabela 1.

**Tabela 1:** Características gerais

	Média (DP)	Frequência (%)
Idade (anos)	53,27 (13,83)	
Feminino		69 (61,6)
Masculino		43 (38,4)
Cateter de Diálise		60 (53,6)
Fistula de Diálise		52 (46,4)
Altura (m)	1,61 (0,10)	
IMC	24,13 (3,97)	
% Gordura Corporal	31,66 (8,44)	
Hemoglobina (g/dL)	12,217 (1,77)	
Hematócrito (%)	37,86 (5,17)	
Plaquetas (/mm^3^)	198500,00 (92511,80)	
Creatinina (mg/dL)	10,09 (2,90)	

A pontuação média na primeira aplicação da DPEBBS foi de 66,97 (DP 7,52) e, na segunda aplicação, de 66,96 (DP 7,71) (Tabela 2).

**Tabela 2:** Consistência interna, concordância e confiabilidade

	Teste	Reteste	Consistência Interna	Concordância		Confiabilidade
	Média (DP)	Média (DP)	α *Cronbach*	SEM	MDD90	ICC (95%)
Pontuação	66,96 (7,52)	66,80 (7,71)	0,885	4,96%	0,02 (0,16)	0,794 (0,73–0,85)
Questão 1	2,87 (0,76)	2,97 (0,80)			–0,01 (–0,09)	
Questão 2	3,14 (0,69)	3,10 (0,74)			0,005 (0,03)	
Questão 3	3,14 (0,59)	3,20 (0,64)			–0,008 (–0,06)	
Questão 4	3,30 (0,61)	3,34 (0,66)			–0,005 (–0,03)	
Questão 5	2,98 (0,79)	2,89 (0,83)			0,01 (0,09)	
Questão 6	3,11 (0,68)	3,16 (0,69)			–0,006 (–0,04)	
Questão 7	3,19 (0,58)	3,26 (0,55)			–0,009 (–0,06)	
Questão 8	1,82 (0,67)	1,79 (0,67)			0,005 (0,03)	
Questão 9	2,58 (0,91)	2,52 (0,94)			0,008 (0,63)	
Questão 10	2,84 (0,78)	2,94 (0,79)			–0,014 (–0,09)	
Questão 11	2,74 (0,78)	2,66 (0,88)			0,012 (0,08)	
Questão 12	2,66 (0,88)	2,48 (0,90)			0,02 (0,18)	
Questão 13	3,16 (0,65)	3,15 (0,67)			0,001 (0,009)	
Questão 14	2,25 (0,85)	2,20 (0,91)			0,006 (0,04)	
Questão 15	2,56 (0,89)	2,51 (0,96)			0,007 (0,04)	
Questão 16	3,27 (0,83)	3,36 (0,58)			–0,01 (–0,09)	
Questão 17	2,75 (0,90)	2,71 (1,00)			0,006 (0,04)	
Questão 18	2,71 (0,89)	2,62 (0,97)			0,01 (0,09)	
Questão 19	2,09 (0,83)	2,02 (0,89)			0,008 (0,06)	
Questão 20	3,13 (0,67)	3,21 (0,69)			–0,01 (–0,08)	
Questão 21	2,55 (0,85)	2,59 (0,90)			–0,005 (–0,03)	
Questão 22	3,14 (0,61)	3,19 (0,64)			–0,006 (–0,04)	
Questão 23	2,55 (0,80)	2,71 (0,88)			–0,02 (–0,15)	
Questão 24	2,33 (0,92)	2,24 (1,00)			0,01 (0,09)	

Abreviaturas – DP: Desvio padrão; SEM: Erro padrão de medição; MDD90: Diferença mínima detectável em 90% de confiança; ICC: Coeficiente de correlação intraclasse; *p*: Significância; R: Coeficiente de correlação de *Pearson*.

A validade de construto foi avaliada por meio da análise das correlações entre o escore total da DPEBBS e os domínios do SF-36. Os coeficientes de correlação de *Spearman* demonstraram associações fracas e predominantemente não significativas entre os domínios do SF-36, incluindo: Capacidade Funcional (ρ = –0,177; p = 0,063), Limitação por Aspectos Físicos (ρ = –0,205; p = 0,031), Limitação por Aspectos Emocionais (ρ = –0,191; p = 0,045), Vitalidade (ρ = –0,059; p = 0,542), Saúde Mental (ρ = –0,076; p = 0,430), Aspectos Sociais (ρ = –0,097; p = 0,311), Dor Corporal (ρ = –0,056; p = 0,559), Estado Geral de Saúde (ρ = –0,050; p = 0,601) e Transição de Saúde (ρ = 0,008; p = 0,933) (Tabela 3).

**Tabela 3:** Validade com cada domÍnio do SF-36

Domínios do SF-36	ρ	Valor de p
Capacidade Funcional	–0,177	0,063
Limitações por Aspectos Físicos	–0,205*	0,031
Limitações por Aspectos Emocionais	–0,191*	0,045
Vitalidade	–0,059	0,542
Saúde Mental	–0,076	0,430
Aspectos Sociais	–0,097	0,311
Dor Corporal	–0,056	0,559
Estado Geral de Saúde	–0,050	0,601
Transição de Saúde	0,008	0,933

Notas – ρ = Coeficiente de correlação de *Spearman*;

* p < 0.05.

As hipóteses de validade convergente e divergente foram testadas por meio da correlação de *Spearman* entre os escores da DPEBBS e os domínios Capacidade Funcional e Estado Geral de Saúde do SF-36. Observou-se uma correlação negativa fraca, porém estatisticamente significativa, com o domínio Capacidade Funcional (ρ = –0,326; p = 0,001), sustentando a validade convergente. Em contrapartida, a correlação com o domínio Estado Geral de Saúde foi fraca e não estatisticamente significativa (ρ = –0,185; p = 0,052), indicando ausência de validade divergente.

Embora a hipótese *a priori* tenha definido a validade convergente como uma correlação negativa fraca (|ρ| < 0,30), a correlação observada com o domínio Capacidade Funcional (ρ = –0,326) foi ligeiramente superior ao esperado. Ainda assim, a magnitude da associação permanece fraca e compatível com a interpretação de construtos relacionados, porém distintos, de acordo com as recomendações do COSMIN.

A análise dos escores do SF-36 revelou os seguintes valores médios (Tabela 4): Capacidade Funcional: 96% (DP 3,38); Limitações Físicas: 42% (DP 0,40); Limitações Emocionais: 47% (DP 0,43); Vitalidade: 53% (DP 0,23); Saúde Mental: 68% (DP 0,23); Aspectos Sociais: 66% (DP 0,31); Dor: 66% (DP 0,29); Estado Geral de Saúde: 45% (DP 0,18); Mudança no Estado de Saúde: 54% (DP 0,27).

**Tabela 4:** Resultados do SF-36

	Pontuação (DP)
Capacidade Funcional	96% (3,38)
Limitações por Aspectos Físicos	42% (0,40)
Limitações por Aspectos Emocionais	47% (0,43)
Vitalidade	53% (0,23)
Saúde Mental	68% (0,23)
Aspectos Sociais	66% (0,31)
Dor Corporal	66% (0,29)
Estado Geral de Saúde	45% (0,18)
Transição de Saúde	54% (0,27)

Abreviaturas – DP: Desvio padrão.

A DPEBBS apresentou alta consistência interna (alfa de *Cronbach* = 0,885), erro de medição muito bom (SEM = 4,96% do escore total) e boa confiabilidade teste-reteste (ICC = 0,794; IC95%: 0,73–0,85), sem efeitos teto ou piso observados. Esses resultados também são demonstrados no gráfico de *Bland–Altman* (Figura 1).

**Figura 1:** Gráfico de *Bland–Altman*

O coeficiente de correlação intraclasse para medidas únicas foi de 0,794 (IC95%: 0,73–0,85), indicando boa confiabilidade dos dados (Tabela 2). Não foram observados efeitos teto ou piso.

## Discussão

A doença renal crônica (DRC) representa um fardo significativo para os sistemas de saúde pública em todo o mundo, com prevalência em crescimento contínuo, especialmente em países de baixa e média renda, como o Brasil^31^. O aumento no número de pacientes incluídos em programas de diálise evidencia a necessidade urgente de estratégias eficazes de manejo voltadas especificamente para essa população^32^. Nesse contexto, o presente estudo teve como objetivo suprir a escassez de instrumentos validados destinados a avaliar níveis de atividade física e barreiras percebidas entre pacientes com DRC em diálise. A *Perceived Exercise Benefits and Barriers Scale for Dialysis Patients* (DPEBBS) foi selecionada para validação devido ao seu potencial de fornecer informações relevantes sobre os fatores que influenciam a participação em atividade física entre pacientes em diálise. Por meio de um rigoroso processo de tradução e adaptação, conduzido de acordo com as diretrizes do COSMIN, foram garantidas a confiabilidade e a validade da escala para uso no contexto brasileiro.

Este estudo enfrentou desafios significativos no processo de tradução e adaptação transcultural de uma escala originalmente desenvolvida em língua chinesa para o português brasileiro. As diferenças linguísticas e culturais entre os idiomas exigiram uma análise semântica minuciosa para assegurar a equivalência dos termos e conceitos. Além disso, a adaptação cultural foi fundamental para garantir que os itens da escala refletissem com precisão as experiências e percepções dos pacientes brasileiros. O processo de validação envolveu um teste piloto e a coleta de feedback de profissionais de saúde e pacientes, resultando em ajustes refinados para aprimorar a compreensão e a aplicabilidade da escala na nova população. A atuação de tradutores com *expertise* em terminologia médica foi essencial para minimizar inconsistências linguísticas e assegurar a uniformidade da tradução em todos os itens da escala. Como resultado desses esforços, obteve-se uma versão traduzida e validada da escala, eficaz e confiável para a avaliação de pacientes brasileiros, contribuindo para uma melhor compreensão e manejo de suas necessidades específicas.

A análise dos dados revelou que o instrumento apresenta características positivas em termos de estabilidade e consistência. As médias dos escores no teste (66,96) e no reteste (66,80) foram bastante semelhantes, indicando boa estabilidade das medidas ao longo do tempo. A consistência interna do instrumento, avaliada pelo alfa de *Cronbach*, foi de 0,885, valor elevado que sugere que os itens são coesos e mensuram consistentemente o mesmo construto. O erro padrão de medição (SEM) foi de 4,96%, indicando baixa variabilidade e elevada confiabilidade dos escores médios. A confiabilidade do instrumento também foi confirmada pela diferença mínima detectável com 90% de confiança (MDD90) entre teste e reteste, que foi de 0,02 (0,16), demonstrando variação mínima nas respostas dos participantes entre as medições e reforçando a estabilidade do instrumento.

O coeficiente de correlação intraclasse (ICC) foi de 0,794, com intervalo de confiança de 95% entre 0,73 e 0,85, confirmando boa concordância entre as medidas e indicando que o instrumento é estável quando aplicado repetidamente aos mesmos indivíduos. Em relação à validade, a maioria das correlações observadas entre a DPEBBS e os domínios do SF-36 apresentou magnitude inferior a 0,3, indicando que os construtos avaliados não são fortemente relacionados. Apenas os domínios “Limitação por Aspectos Físicos” e “Limitação por Aspectos Emocionais” apresentaram correlações negativas fracas e estatisticamente significativas, ainda assim classificadas como construtos não relacionados, de acordo com a classificação do COSMIN.

Na análise dos itens específicos do instrumento, as respostas médias apresentaram variações discretas entre o teste e o reteste, enquanto as correlações permaneceram estáveis, indicando elevada consistência e estabilidade das respostas. Em síntese, as análises estatísticas demonstraram boa consistência interna, confiabilidade e validade convergente, bem como validade divergente adequada. Esses achados reforçam a robustez do instrumento para avaliar os construtos a que se propõe.

Os dados descritivos dos participantes fornecem uma visão abrangente das características demográficas e clínicas da amostra. A idade média foi de 53,27 (±13,83) anos, com índice de massa corporal médio de 24,13 (±3,97), valores compatíveis com populações típicas de pacientes em hemodiálise, apesar das variações demográficas observadas^33,34^. A análise por sexo revelou maior prevalência de mulheres (61,6%), aspecto que deve ser considerado na generalização dos resultados para outras populações^35^. Os parâmetros bioquímicos apresentados (como hemoglobina, hematócrito, creatinina, ureia, potássio, fósforo, cálcio, sódio, ferritina e ferro) situam-se dentro dos valores esperados para pacientes em hemodiálise, refletindo a complexidade e os desafios inerentes ao manejo da DRC^36^. O número médio de hospitalizações nos últimos anos constitui um indicador relevante da carga da doença, com média de 0,31 (±0,54) internações no último ano e 2,15 (±1,93) nos últimos cinco anos, evidenciando a necessidade de intervenções contínuas para a manutenção da saúde desses pacientes^37,38^.

A análise dos escores do *Short Form Health Survey 36* (SF-36) evidenciou prejuízos importantes em diversos domínios da qualidade de vida relacionada à saúde. Limitações físicas, limitações emocionais e redução da vitalidade foram particularmente expressivas, destacando o impacto multifacetado da DRC sobre o bem-estar dos pacientes^39^. Esses achados reforçam a importância de abordar não apenas barreiras físicas, mas também fatores psicossociais que podem influenciar a adesão à atividade física em pacientes em diálise.

Em comparação com o estudo original de Zheng et al., a população avaliada neste estudo apresentou diferenças que podem ter influenciado os valores de validade observados. No estudo original, a maioria dos participantes era do sexo masculino e o principal acesso dialítico era a fístula arteriovenosa, enquanto, no presente estudo, predominou o sexo feminino e o uso de cateter como principal forma de acesso à hemodiálise^22^.

No que se refere à relação entre qualidade de vida e percepção de barreiras e benefícios da atividade física, os resultados do presente estudo foram semelhantes aos de Zheng et al., sendo a baixa qualidade de vida um achado recorrente na população avaliada. Ainda assim, os participantes reconheceram os benefícios da atividade física, sugerindo que a baixa adesão a programas de exercício e reabilitação pode estar associada a outros fatores, como fadiga ou medo de lesões^9^. Os índices psicométricos observados para a DPEBBS neste estudo também são comparáveis aos relatados em investigações anteriores que utilizaram a DPEBBS e outros instrumentos de desfecho relatado pelo paciente em populações em hemodiálise^12^. No estudo de Ghafourifard et al., a DPEBBS apresentou consistência interna satisfatória e associações relevantes com desfechos autorreferidos, apoiando seu uso em pesquisas clínicas. De forma semelhante, a DPEBBS demonstrou alta consistência interna, boa confiabilidade teste-reteste e erro de medição aceitável, indicando escores estáveis e reprodutíveis ao longo do tempo. Esses índices de confiabilidade são comparáveis aos relatados para instrumentos amplamente utilizados no cuidado ao paciente em diálise, como o KDQOL-SF *(Kidney Disease Quality of Life – Short Form)* e o SF-36*,* que, embora avaliem construtos mais amplos e não específicos de exercício, apresentam consistência interna e reprodutibilidade adequadas. Assim, a DPEBBS demonstra desempenho psicométrico comparável ao de escalas consolidadas no contexto da diálise, com a vantagem adicional de captar especificamente a percepção dos pacientes sobre benefícios e barreiras relacionados ao exercício físico, um construto diretamente relevante para o planejamento e a avaliação de intervenções de atividade física em hemodiálise.

Os achados do presente estudo são amplamente consistentes com os relatados por Lightfoot et al. (2021)^14^, que investigaram percepções de benefícios e barreiras ao exercício em uma grande amostra de pacientes em hemodiálise e diálise peritoneal utilizando a DPEBBS. Em ambos os estudos, a fadiga emergiu como uma das barreiras mais frequentemente relatadas, confirmando seu papel central como fator limitante para a prática de exercícios nessa população. De forma semelhante, benefícios relacionados à melhoria da qualidade de vida, do humor e da função física foram amplamente reconhecidos pelos pacientes em ambos os contextos, sugerindo uma percepção consistente dos efeitos positivos do exercício. No entanto, diferenças na frequência de determinadas barreiras, como aquelas relacionadas às comorbidades e ao impacto do exercício na vida familiar, foram observadas entre os estudos, possivelmente refletindo variações nos perfis clínicos, nos cenários de cuidado e nos desenhos metodológicos. Apesar dessas diferenças, os padrões gerais de resposta indicam que pacientes em diálise compartilham percepções semelhantes quanto aos principais benefícios e obstáculos à prática de exercícios, reforçando a necessidade de estratégias de intervenção que abordem especificamente a fadiga e enfatizem benefícios considerados clinicamente relevantes pelos próprios pacientes.

Wingood et al. destacaram a considerável heterogeneidade na avaliação das barreiras percebidas à atividade física entre adultos com 50 anos ou mais, enfatizando a necessidade de instrumentos específicos para diferentes populações^21^. Embora 33 ferramentas distintas tenham sido identificadas na revisão, a maioria foi validada em apenas um estudo, e nenhuma foi desenvolvida especificamente para pacientes em diálise. Nesse contexto, o presente estudo preenche uma lacuna importante ao disponibilizar uma versão brasileira validada de um instrumento direcionado à população em diálise. Diferentemente de escalas genéricas, como a *Exercise Benefits/Barriers Scale* (EBBS), a DPEBBS aborda benefícios e barreiras diretamente relacionados ao contexto do tratamento dialítico, o que pode explicar as correlações fracas, porém teoricamente coerentes, observadas com os domínios do SF-36. Esses resultados reforçam a compreensão de que instrumentos focados em barreiras ao exercício avaliam construtos relacionados, porém distintos, da qualidade de vida geral e destacam a importância de avaliações psicométricas robustas em populações clínicas específicas, especialmente em países de baixa e média renda com elevada carga de DRC.

Pesquisas futuras devem concentrar-se na avaliação da responsividade da DPEBBS em ensaios de reabilitação em hemodiálise, particularmente em estudos voltados à promoção da atividade física e da adesão ao exercício. Investigações longitudinais também são necessárias para estabelecer pontos de corte clinicamente significativos, como escores associados à baixa participação em exercícios ou à adesão insatisfatória a programas de reabilitação. Além disso, a validação de versões alternativas da escala, incluindo formatos eletrônicos e adaptações para outros idiomas, poderá ampliar ainda mais sua aplicabilidade em diferentes contextos clínicos e culturais.

### Implicações na Prática da Fisioterapia

Os achados deste estudo sugerem que a DPEBBS pode ser incorporada à prática clínica como uma ferramenta de suporte à tomada de decisão, especialmente no contexto da reabilitação de pacientes em hemodiálise. Sua aplicação permite identificar perfis de pacientes com maior percepção de barreiras à atividade física, favorecendo a estratificação de risco comportamental e a priorização de intervenções.

Adicionalmente, o instrumento pode subsidiar o desenvolvimento de estratégias individualizadas de exercício, alinhadas às necessidades e percepções dos pacientes, contribuindo para abordagens mais centradas no paciente. Nesse sentido, seu uso pode otimizar o planejamento terapêutico, especialmente em programas intradialíticos ou domiciliares, ao integrar aspectos físicos e psicossociais relacionados à adesão ao exercício.

### Limitações

Este estudo apresenta limitações que devem ser consideradas na interpretação dos resultados. Primeiramente, trata-se de um estudo conduzido em um único centro, o que pode restringir a generalização dos achados para outras populações e contextos assistenciais.

Além disso, a predominância de participantes do sexo feminino pode ter influenciado os resultados, considerando possíveis diferenças na percepção de barreiras e benefícios relacionados à atividade física.

A utilização do SF-36 como medida de validade de construto, embora amplamente aceita, pode não capturar de forma específica aspectos relacionados ao exercício físico, o que pode justificar as correlações de baixa magnitude observadas.

Adicionalmente, a ausência de avaliação da responsividade limita a compreensão da capacidade do instrumento em detectar mudanças ao longo do tempo. Por fim, a não realização de análise fatorial exploratória ou confirmatória restringe a verificação da estrutura dimensional da escala na população brasileira.

## Conclusão

A versão brasileira recebeu a nomenclatura “Escala de Percepção da Atividade Física em Diálise (EPAD)”.

A EPAD demonstrou adequada validade e confiabilidade para uso em pacientes em diálise, configurando-se como um instrumento robusto para a avaliação de benefícios e barreiras percebidas à prática de atividade física. Apesar de suas propriedades psicométricas satisfatórias, limitações como a realização do estudo em um único centro devem ser consideradas na interpretação dos resultados. Ainda assim, a EPAD apresenta potencial aplicabilidade clínica, podendo subsidiar o aconselhamento individualizado e o planejamento de intervenções de exercício em unidades de hemodiálise, contribuindo para estratégias mais centradas no paciente.

## Data Availability

Os dados que sustentam os achados deste estudo estão disponíveis com o autor correspondente, mediante solicitação razoável.

## Material Suplementar

O seguinte material online está disponível para o presente artigo:

Versão em português brasileiro da DPEBBS.
